# Which casein micelle removal method is suitable for studies of human milk extracellular vesicles? A systematic comparison of four different treatments for casein depletion before extracellular vesicle isolation from human milk

**DOI:** 10.20517/evcna.2024.02

**Published:** 2024-05-22

**Authors:** Hatice Cetinkaya, Supasek Kongsomros, Laurie Nommsen-Rivers, Ardythe L. Morrow, Somchai Chutipongtanate

**Affiliations:** ^1^MILCH and Novel Therapeutics Lab, Division of Epidemiology, Department of Environmental and Public Health Sciences, University of Cincinnati College of Medicine, Cincinnati, OH 45267, USA.; ^2^Department of Nutrition, University of California Davis, Davis, CA 95616, USA.; ^#^Authors contributed equally.

**Keywords:** Acidification, casein micelles, calcium chelation, coagulation, extracellular vesicles, human milk, method comparison

## Abstract

**Aim:** This study aimed to systematically compare four casein micelle removal methods on the particle and protein characteristics of the isolated human milk EVs.

**Methods:** The defatted milk was treated with 1% sodium citrate, 20 mM ethylenediaminetetraacetic acid (EDTA), 1% acetic acid, or 1% chymosin/calcium chloride for 30 min at 4 °C to remove casein micelles. EV isolation was performed using qEV size exclusion chromatography. Milk turbidity at the optical density 350 nm and dot immunoblot with casein antibody were applied to monitor the qEV fractions. Particle analyses were performed using transmission electron microscopy (TEM) and nanoparticle tracking analysis (NTA). The enrichment of human milk EV markers, i.e., tetraspanins, Alix, lactadherin, butyrophilin, and xanthine dehydrogenase, and casein depletion capabilities were evaluated by proteomics and immunoblotting.

**Results:** Compared to the untreated condition, sodium citrate and EDTA decreased milk turbidity by disrupting casein micelles, while acetic acid and chymosin removed them by inducing precipitation/coagulation. All treatments shifted casein immunoreactivity in the qEV fractions from large micelles (the exclusion volume) to small molecular sizes (gel-infiltrated fractions). Acidification affected human milk EV morphology, while EDTA, acetic acid, and chymosin methods slightly altered EV particle numbers. Different casein micelle removal methods confer different degrees of human milk EV marker enrichment and casein depletion. The method performances could be ranked as follows: chymosin > EDTA > acetic acid > sodium citrate.

**Conclusion:** Our findings suggest that chymosin and EDTA should be considered as the method of choice for casein micelle removal in future studies involving human milk EV isolation and characterization.

## INTRODUCTION

Extracellular vesicles (EVs) in human milk primarily originate from mammary epithelial cells. Human milk-derived EVs (HMEVs) transfer proteins, lipids, and nucleic acids and play a central role in maternal-child biochemical communication^[1]^. Interest in understanding the fundamental biology and potential applications of HMEVs has been growing^[1-6]^. Despite the rising number of publications regarding HMEVs, an optimal standard method to isolate EVs from human milk is lacking. According to the global survey conducted by the International Society for Extracellular Vesicles (ISEV) Rigor and Standardization Subcommittee in 2021^[7]^, techniques to isolate the EVs from different biofluids, including human milk, can vary across the research groups, depending on the rationale and study objectives. Nonetheless, in the context of a complex human milk matrix, which contains a high abundance of milk fat globules, cells, soluble proteins, and casein micelles, preliminary steps should be performed to remove potential contamination before HMEV isolation. Milk fat globules and cells can be removed from whole milk by centrifugation^[5]^, and soluble proteins can be separated to varying extent from HMEV isolates by most EV isolation methods^[8]^. A significant challenge for HMEV isolation, however, is removing casein micelle contamination due to its abundance in human milk.

Caseins, comprising alpha-, beta- and kappa-isoforms, are a major protein component (3.6 g/L; about 40% of total protein) in human milk^[9,10]^. Caseins interact with colloidal calcium phosphate to form the supramolecular structure of casein micelles^[11]^. The biophysical size of casein micelles (100-600 nm)^[12]^ overlaps with EVs (small EVs < 200 nm; large EVs 200-1,000 nm)^[10,11]^. Moreover, casein micelles have a density of 1.06 g/mL^[10]^, while EVs exhibit a density of 1.08-1.19 g/mL^[10,13]^. As a result, casein micelles and HMEVs (especially small EV subpopulations) are almost always co-isolated by most isolation techniques. Removing or disaggregating casein micelles in human milk prior to HMEV isolation is therefore a critical strategy for improving the purity of HMEV.

Several methods have been applied to disaggregate and remove casein micelles from human milk. These include acidification^[14,15]^, calcium chelators by sodium citrate^[16]^, ethylenediaminetetraacetic acid (EDTA)^[17]^, and chymosin^[18,19]^. The mechanisms by which casein micelles are removed vary across these methods. For example, sodium citrate and EDTA chelate calcium to disrupt the supramolecular structure of casein micelles down to casein sub-micellar monomers (< 40 nm)^[16,17]^. Acidification of milk disaggregates casein micelles by dissolving calcium phosphate and induces isoelectric precipitation of caseins^[14,15,20]^. Chymosin is a protease that hydrolyses the negatively charged end of kappa-caseins of the micelles, causing destabilization and coagulation of casein micelles^[18,19]^. Note that the substrate specificity of chymosin differs slightly between bovine and human kappa caseins; bovine kappa casein is cleaved at the peptide bond between amino acid residue 126-127 (phenylalanine-methionine; P02668, uniprot.org) while human kappa casein is cleaved at amino acid residue 117-118 (phenylalanine-isoleucine; P07498, uniprot.org).

Human milk differs from bovine milk regarding physicochemical characteristics, including the composition and amounts of casein micelles^[9,21-23]^. Bovine milk composition is high in alpha-casein (greater than 50% of whole casein)^[24]^, while human milk composition is high in beta-casein (approximately alpha 15%, beta 55%, kappa 30% at 2-week lactation)^[22]^. Furthermore, alpha- and beta-casein have different biochemical properties; alpha-casein is more hydrophilic, while beta-casein is more hydrophobic^[25]^. These casein differences may affect the performance of casein removal methods that are applied to bovine milk and human milk.

Previous studies have compared various methods for removing casein micelles, particularly for isolating EVs from bovine milk^[15,17,26]^. However, research on casein micelle removal methods specifically for EV isolation from human milk is lacking. The physicochemical differences between human and bovine milk suggest that methods effective for bovine milk may or may not be directly applicable to human milk. Therefore, this study aimed to systematically compare and optimize methods previously used on bovine milk, including sodium citrate, EDTA, acetic acid, and chymosin treatments for casein removal, adapting these methodologies to the distinct isolation of HMEVs. This paper represents more than incremental progress by establishing an optimized, human milk-specific method for EV isolation from clinical samples.

## MATERIALS AND METHODS

### Human milk sample preparation

Whole milk samples from four donors were retrieved from the MOM2CHild Study (IRB protocol ID: 2022-0653) and were pooled to make a stock of 20 mL. Milk samples were collected at week 2 postpartum. Written informed consent to participate was obtained. The milk samples were centrifuged at 3,000 × *g* for 10 min at 4 °C to remove milk fat and cell pellets and then centrifuged at 12,000 × *g* for 20 min at 4 °C to remove large vesicles and aggregates. The resulting defatted milk samples were aliquoted and stored at -80 °C.

### Casein micelle removal

The defatted milk (1 mL) was treated with sodium citrate (1% final; S4641, Sigma, USA), EDTA (20 mM final; E6758, Sigma, USA), acetic acid (1% final; A6283, Sigma, USA), and chymosin/ calcium chloride (CaCl_2_) (1% final; # 50-111-8061, Thermo Fisher Scientific, USA) and incubated at 4 °C for 30 min. After incubation, the samples with sodium citrate and EDTA were ready for EV isolation. In contrast, the samples treated with acetic acid and chymosin required an additional step of centrifugation at 12,000 × *g*, 4 °C for 30 min to remove casein precipitates before EV isolation.

To measure the kinetic of milk turbidity changes, the defatted milk was treated with sodium citrate (1% final), EDTA (20 mM final), acetic acid (1% final), and chymosin/CaCl_2_ (1% final) and then serially measured with the optical density at 350 nm at 0, 0.5, 1, 2, 5, 10, 15, and 30 min using the Agilent BioTek synergy microplate reader. This experiment was performed in three independent replicates.

### HMEV isolation

For EV isolation, the samples were concentrated for large molecules while removing low molecular weight contamination using 100-kDa cutoff centrifugal filtration (Merck Millipore). The concentrated samples (0.5 mL) were then loaded onto an IZON Gen-2 35 nm qEV size-exclusion column (Izon Science, UK) to isolate EVs from soluble proteins. A total of 20 fractions (0.5 mL each) were collected and measured by the optical density at 350 nm to identify the particle elutes using the Agilent BioTek synergy microplate reader. The EV isolates were aliquoted and kept at -80 °C until used.

### Detection of caseins in EV fractions using dot immunoblot

Two microliters of each fraction (1 to 20) were blotted onto the nitrocellulose membrane. After air drying for 30 min, the membrane was then blocked with Everyblot blocking buffer (Bio-Rad, USA) at room temperature for 5 min and stained with the primary antibody of β-casein antibody (sc-53189; Santa Cruz Biotechnology, Inc., Dallas, TX) at 1:500 dilution in Everyblot blocking buffer at 4 °C overnight. After washing, the membranes were incubated with goat anti-mouse IgG secondary antibody-HRP (Invitrogen) at room temperature for 1 h. The dot blot was developed by SuperSignal West Pico PLUS Chemiluminescent (ThermoFisher) and imaged on a ChemiDoc Touch Imaging system (Bio-Rad, USA).

### Nanoparticle tracking analysis

NanoSight NS300 was used with an integrated sample pump (Malvern Instruments Ltd., Malvern, Worcestershire, UK). The EV isolates were diluted at 1:100 in 0.22 µm filtered phosphate buffer saline (PBS) to 1 mL. The sample was then injected into the chamber with a syringe pump. Five 1-min videos were captured for each sample with the following parameters: camera: sCMOS; cell temperature: 25 °C. After capture, the videos were analyzed by NanoSight Software NTA 3.4 Build 3.4.003 with this setting: detection threshold: 5; blur size and max jump distance: auto. Ideal concentrations were measured at 20-100 particles/frame.

### Transmission electron microscopy

Five microliters of the EV isolates were applied onto a 200-mesh grid (FCF200-CU; Electron Microscopy Sciences) and negatively stained by using 2% uranyl acetate (#22400; Electron Microscopy Sciences) for 10 seconds. The grid was then washed 2 times with deionized water before air drying for 1 minute at room temperature. Imaging was performed using a transmission electron microscope (Thermo Talos L120C Transmission Electron Microscope) with an acceleration voltage of 100 kV.

### Western blot analysis

The EV sample (5 μg protein) was lysed with RIPA buffer and mixed with 4x Leammli buffer (Biorad, USA). The samples were heated at 95 °C for 5 min and then run into the NuPAGE™ 12%, Bis-Tris, 1.0 mm, mini protein gels for 50 min (Invitrogen, USA) before being transferred onto a PVDF membrane using a transblot (Bio-Rad, USA). The membrane was blocked with Everyblot blocking buffer (Bio-Rad, USA) and then probed with 1:1,000 rabbit anti-CD9 (ab223052) or β-casein antibody (sc-53189; Santa Cruz Biotechnology, Inc.) at 1:500 at 4 °C overnight. After washing, the membranes were incubated with 1:3,000 goat anti-rabbit IgG secondary antibody HRP (Abcam) (for CD9) and goat anti-mouse IgG secondary antibody HRP (Invitrogen) (for β-casein) at room temperature for 1 h. The immunoblot was developed by SuperSignal West Pico PLUS Chemiluminescent (ThermoFisher) and imaged on a ChemiDoc Touch Imaging system (Bio-Rad, USA).

### Mass spectrometric proteomics

Proteomic analysis was performed as described previously^[5]^. Briefly, two micrograms of EV proteins were reduced, alkalinized, and digested by trypsin (1:50 w/w; MS-grade, Thermo) at 37 °C overnight, and cleaned up by C18 solid phase extraction. Data were collected on an Orbitrap Eclipse mass spectrometer coupled to Dionex Ultimate 3000 RSLCnano (Thermo) using an EASY-Spray column PepMap RSLC C18 with a 150-mm column (i.d. 75 m, C18, 3.0 m, 100 Å) with a mobile phase gradient from 98% phase A (0.1% formic acid/H_2_O) to 32% phase B (0.1% formic acid/ACN) for 60 min at 300 nL/min. For Data-Dependent Acquisition (DDA), MS1 was collected in Orbitrap (120,000 resolution; maximum injection 50 ms; automatic gain control [AGC] 4 × 10^5^). Charge states between 2 and 6 were required for MS2 analysis with a 20-s dynamic exclusion window and cycle time of 2.5 s. MS2 scans were performed in an ion trap with higher-energy collisional dissociation (HCD) fragmentation (isolation window 0.8 Da; normalized collision energy (NCE) 30%; maximum injection 40 ms; AGC 5 × 10^4^). For BoxCAR, MS1 was collected in Orbitrap (120,000 resolution; maximum injection 50 ms; normalized AGC target 300%, scan range 400-1,200 m/z, RF lens 40%). Charge states between 2 and 6 were required for MS2 analysis with a 25-s dynamic exclusion window. MS2 scans were performed in two BoxCAR scans (15,000 resolution; isolation window 1.4 m/z; fixed NCE 30%; normalized AGC target 100%; maximum injection time 20 ms). Data were recorded using Xcalibur 4.3 (Thermo). The raw files were searched by MaxQuant software with default search parameters against the human protein database to identify and quantify peptides at FDR < 1%. Raw data were normalized by the total area sum (TAS) method^[27]^. Only proteins identified and quantified by two peptides and consistently detected by DDA and BoxCAR were included in the analysis.

## RESULTS

Casein micelles share a comparable particle diameter with EVs^[10-12]^, have a very high abundance in milk matrix, and are the significant contamination during HMEV isolation. This study, therefore, compared four biochemical methods commonly applied to remove caseins from bovine and human milk in literature and measured particle and protein evidence following HMEV isolation.

### Chymosin treatment increased milk turbidity by agglutinating casein micelles, while acetic acid, EDTA, and sodium citrate decreased turbidity by disrupting the micelles

**Figure 1:**
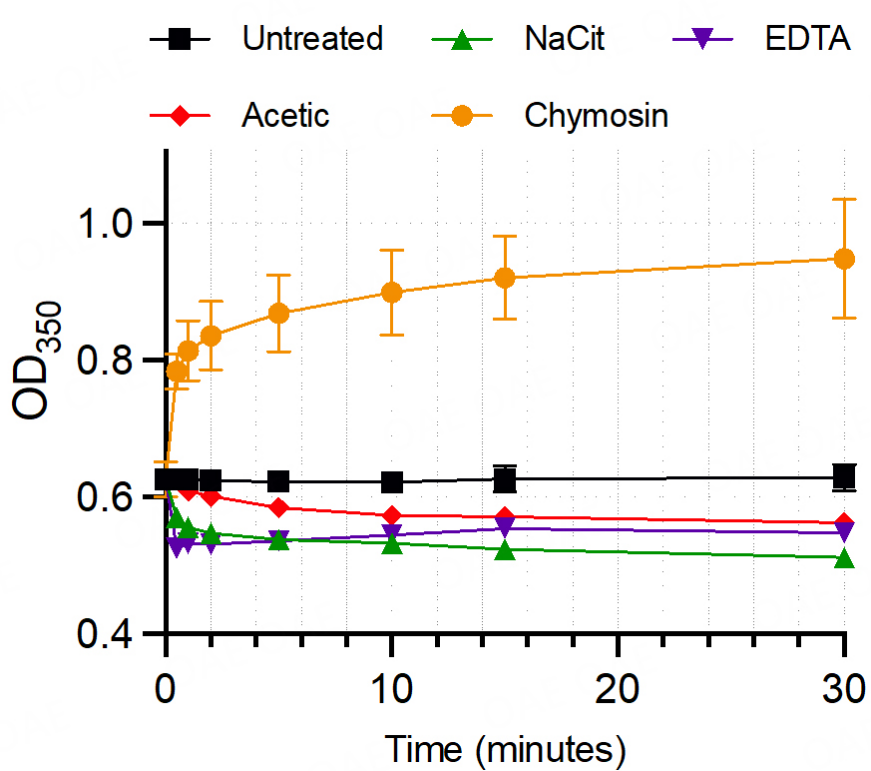
The kinetics of milk turbidity changes upon different casein micelle removal treatments. The defatted milk was treated with sodium citrate (NaCit), EDTA, acetic acid, and chymosin/CaCl_2_. The mixture was measured with the OD_350_ at 0, 0.5, 1, 2, 5, 10, 15, and 30 min following treatments (*n* = 3 independent replicates). The results were reported as mean ± standard error. EDTA: ethylenediaminetetraacetic acid.

To assess the impact of different casein micelle disaggregation/removal methods, we measured the kinetic of milk turbidity changes following treatments with 1% sodium citrate, 20 mM EDTA, 1% acetic acid, and 1% chymosin/CaCl_2_ at 0, 0.5, 1, 2, 5, 10, 15, and 30 min. The optical density at 350 nm (OD_350_) was applied as this wavelength is sensitive to monitor the eluted EVs from size exclusion chromatography. Figure 1 shows that chymosin treatment gradually increased the OD_350_ values over time compared to the untreated condition, suggesting increased turbidity, which is consistent with its mechanism to agglutinate casein micelles. In contrast, calcium chelators, including EDTA and sodium citrate, markedly decreased the OD_350_ values by disrupting the casein micelle complexes, reaching the plateau within 10 min of incubation. Acetic acid treatment gradually reduced the OD_350_ values over time by changing the solution pH, resulting in casein precipitation out of the solution. Note that both chymosin and acetic acid methods required an incubation time of up to 30 min before the OD_350_ reached the plateau and needed an additional step of centrifugation to remove the casein aggregates before submitting the supernatant for HMEV isolation, thus increasing the workload and turnaround time compared to EDTA and sodium citrate treatments.

### All casein micelle removal treatments shift casein immunoreactivity from large micelles to lower molecular sizes, as detected by a dot blot of qEV fractions

**Figure 2:**
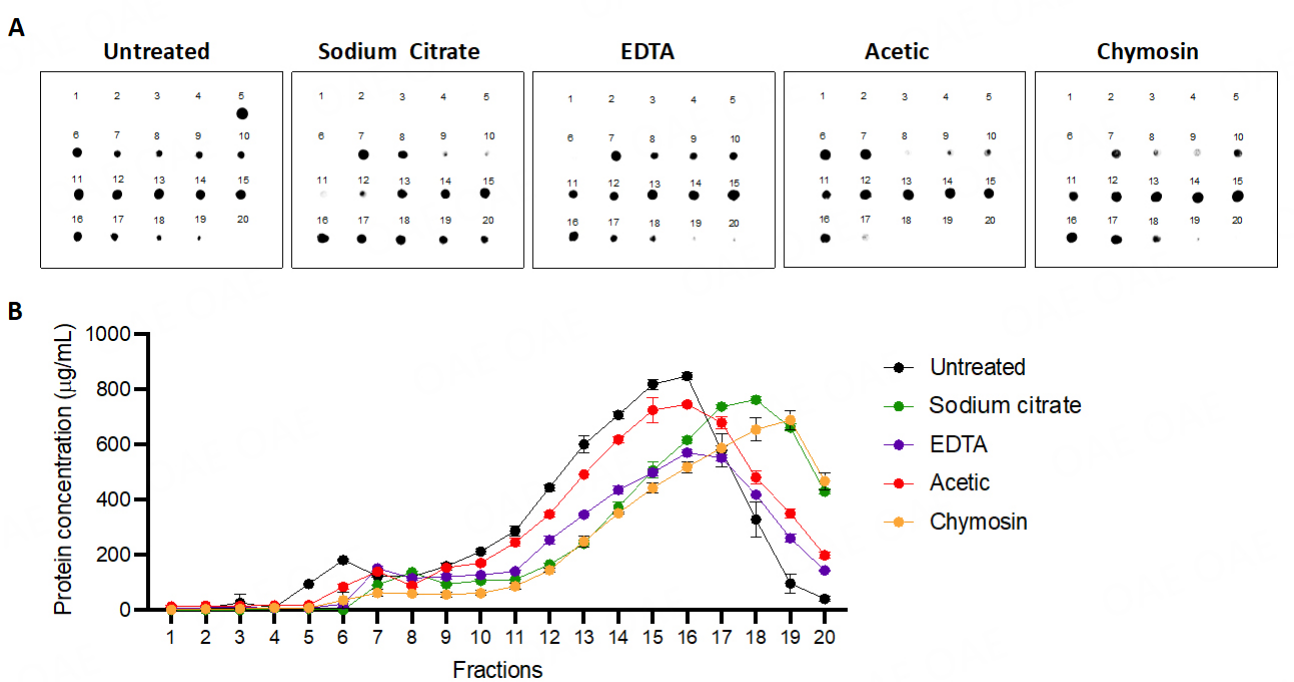
Dot blot analysis of qEV fractions using anti-casein antibody. (A) The representative dot blot images display the profiles of milk EV-isolated fractions after treatment with casein micelle disaggregation/removal reagents. Two microliters of each of the 20 fractions were blotted onto a nitrocellulose membrane. The membranes were then probed with antibodies specific for β-casein and developed using anti-mouse-HRP for signal detection; (B) Protein concentrations in all collected fractions measured by Pierce 660 protein assay. EDTA: ethylenediaminetetraacetic acid.

To demonstrate the capability of casein micelle removal based on the biophysical property, this study performed qEV size exclusion chromatography, which allowed us to separate the supramolecular structure of casein micelles (eluted at the exclusion volume of the qEV column similar to HMEVs; fractions 5 and 6) from sub-micellar caseins and their monomers that have smaller biophysical sizes (eluted at fractions 7-20). Following the qEV fractionation, casein antigenicity in every fraction (from 1 to 20) was detected by dot immunoblot with casein-specific antibody [Figure 2A]. The results showed that: (i) in the untreated condition, high casein immunoreactive signals were observed in fractions 5 and 6 (representing casein micelles) with tails in fractions 7-10 (representing sub-micellar caseins) and fractions 11-15 (indicating casein monomers); (ii) all casein micelle removal methods could shift the firstly detected fraction of casein immunoreactivity from the qEV exclusion volume, suggesting depletion of casein micelle contaminants in the collected HMEVs (fractions 5 and 6); (iii) by visual recognition, the method efficacy in shifting the supramolecular structure of casein micelles to smaller casein monomers which could infiltrate into the qEV gel beads were as follows: chymosin > EDTA > sodium citrate > acetic acid. Consistently, the protein content of all the collected fractions recapitulated the shift of casein micelles to the smaller forms in the later fractions [Figure 2B]. This finding strongly suggested that all treatments can reduce casein micelles from the EV fractions. Of note, dot immunoblot can only detect caseins in the solution, not caseins inside the lipid bilayer-enclosed HMEVs.

### Casein micelle removal methods affect the intact HMEV morphology and particle size distribution differently

**Figure 3:**
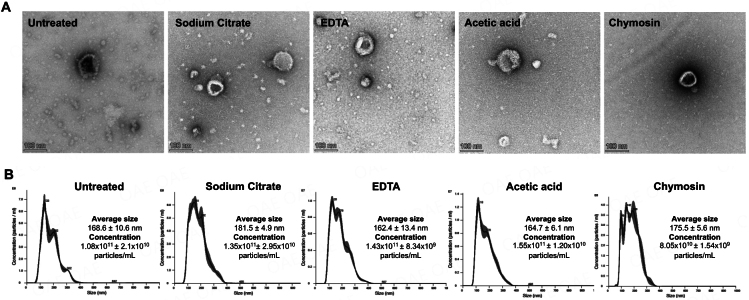
HMEV particle evidence after casein micelle removal treatment. (A) Transmission electron microscopy (TEM) images show the cup-shaped morphology of isolated EVs following casein micelle disaggregation/removal; (B) Nanoparticle tracking analysis (NTA) presents the size distribution, average diameter, and concentration (particles/mL) of the HMEVs following each treatment. EDTA: ethylenediaminetetraacetic acid.

To determine whether casein micelle removal methods altered EV particle characteristics, TEM was used to visualize the single vesicle morphology, and NTA was applied to determine particle size distribution. As a result, TEM exhibited HMEVs as cup-shaped membrane-enclosed nanoscale vesicles. Their shape and morphology were not affected by most methods except acidification which may induce the rough surface morphology [Figure 3A]. NTA showed that EDTA and acetic acid methods slightly increased the number of particles and chymosin slightly decreased the EV numbers compared to the untreated condition [Figure 3B]. One possible explanation is that EDTA and acetic acid methods may release a number of EVs from casein micelle complexes while chymosin, because of its mechanism to induce casein micelle coagulation, may trap and remove some of EVs during aggregation removal by centrifugation. These findings suggested that different casein removal methods may affect HMEV morphology and particle numbers.

### Different casein micelle removal methods confer different degrees of HMEV protein marker enrichment and casein depletion

It was expected that, after casein micelle removal, the HMEVs and their associated protein markers would be enriched. In this direction, we conducted proteomic analyses to characterize common EV proteins^[8]^ and specific HMEV markers^[28,29]^ and assess the amounts of casein contamination in the HMEV isolates. As a result, proteomic analysis detected 393 proteins across all treatment groups (details in Supplementary Table 1), while 87 proteins were consistently detected in all samples by two different proteomic acquisition methods (DDA and BoxCAR; details in the Methods section and Supplementary Table 2). A Venn diagram of proteomic data was provided in Supplementary Figure 1 and Supplementary Table 3. As expected, most proteins were shared among all biochemical methods. One obvious change was that all casein removal methods increased the number of identified proteins compared to the untreated condition. This finding is consistent with the fact that the biochemical methods remove a substantial amount of casein from the sample, thereby increasing the ability of mass spectrometry to detect the lower abundant proteins in the EV isolates.

**Figure 4:**
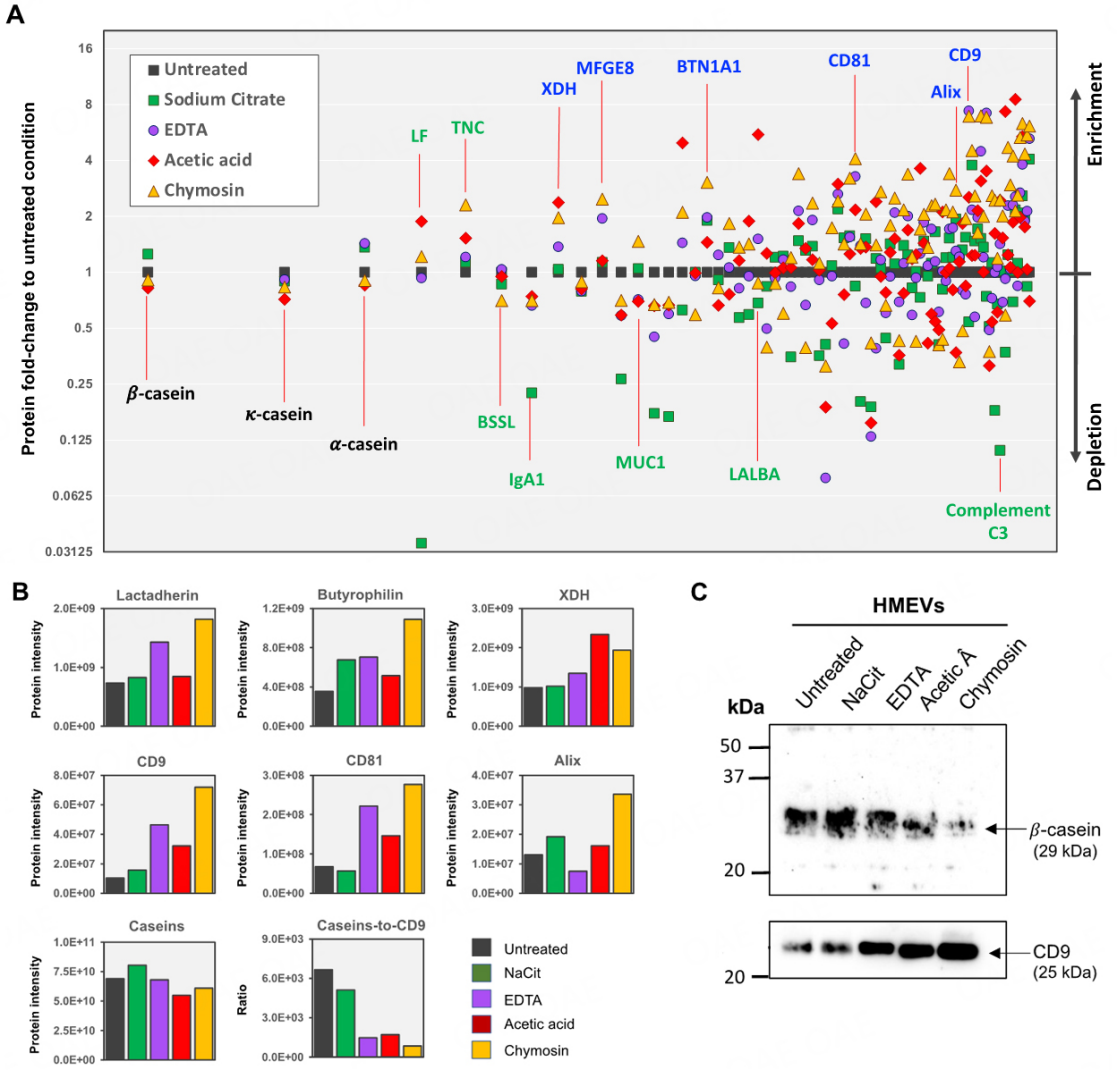
HMEV protein evidence after casein micelle removal treatment. Proteomic analysis of HMEVs isolated after casein micelle disaggregation/removal by sodium citrate, EDTA, acetic acid, and chymosin treatments [full results in Supplementary Table 1]. (A) Fold-changes of 87 HMEV proteins as compared to the untreated condition (details in Supplementary Table 2). Color codes: blue, common and specific HMEV markers; green, highly abundant soluble proteins in human milk; black, caseins; (B) The average protein intensities of selected HMEV markers, total caseins (alpha-, beta-, kappa-caseins), and the ratio of caseins per CD9; (C) Western immunoblot demonstrated that different casein micelle removal methods exhibited different capabilities of casein depletion and HMEV enrichment (full-length blots of the cropped images were provided in Supplementary Figure 2). BTN1A1: butyrophilin 1A1; BSSL: bile salt-stimulated lipase; LALBA: alpha-lactalbumin; LF: lactoferrin; MFGE8: milk fat globule-epidermal growth factor 8 (also known as lactadherin); MUC1: mucin 1; NaCit: sodium citrate; TNC: tenascin; XDH: xanthine dehydrogenase. EDTA: ethylenediaminetetraacetic acid.

Figure 4A demonstrates the fold changes of 87 HMEV proteins following casein micelle removal methods compared to the untreated condition. In contrast, Figure 4B illustrates the relative protein intensities of selected EV markers and caseins. Overall, the result showed that: (i) the common EV proteins and HMEV-specific markers (labeled in blue) were enriched; (ii) several highly abundant milk proteins (labeled in green) such as IgA, alpha-lactalbumin (LALBA) and complement C3 were depleted; (iii) in contrast, a few highly abundant milk proteins, including lactoferrin (LF) and tenascin (TNC), were slightly enriched; (iv) interestingly, three isoforms of caseins (labeled in black) were slightly reduced in the HMEV isolates following casein micelle removal. To validate these protein expression data, we performed Western immunoblot to determine the amounts of beta-casein and CD9 marker in the HMEV isolates after casein micelle removal. As expected, Western blot results were consistent with proteomic findings [Figure 4C] and yielded an insight into the method performance for depleting caseins and enriching HMEVs.

## DISCUSSION

This study systematically compared four biochemical methods and their performance in reducing casein contamination while enriching HMEVs isolated from human milk. Comparisons included sodium citrate and EDTA, which disaggregate casein micelles by calcium chelation, and acetic acid and chymosin/CaCl_2_, which precipitate caseins out of the solution. The main findings are that all methods could remove or disaggregate large casein micelles out of the qEV exclusion volume [Figure 2]; different methods had different effects on HMEV particle analysis and yields [Figure 3]; and that different methods had different efficacy on casein depletion and HMEV enrichment [Figure 4].

The strength of calcium chelation-based methods is their simplicity and turnaround time. Both sodium citrate and EDTA treatments need only one step of the procedure to mix the chemicals with the human milk matrix, and casein micelle disaggregation reaches a plateau within 10 min [Figure 1]. After ten min, the mixture is ready for HMEV isolation. In contrast, casein precipitation/coagulation methods require a two-step procedure: first, sample treatment and second, centrifugation. In our experience, the optimal incubation time for acetic acid and chymosin treatments can be up to 30 min [Figure 1]. The workload and turnaround time difference between calcium chelation-based and casein precipitation methods may be insignificant in small projects, but the difference in efficiency is an important consideration in larger studies.

In our study, HMEV morphology was not affected by most casein removal treatments, which was consistent with the findings of Tong *et al.* in their comparison of chymosin and HCl treatment^[26]^. On the other hand, two studies reported a rough surface of bovine milk EVs treated with acidification despite the vesicle morphology remaining spherical^[15,30]^. In our experiments, we used human milk samples rather than bovine milk. Our TEM results showed that the acetic acid method may also induce the rough surface of HMEVs [Figure 3A], even though that change was not as obvious as reports from previous studies of bovine EVs^[15,30]^. Human milk features lower concentrations of casein, lower levels of alpha- and higher beta-casein isoforms, smaller casein micelles, and exhibits less calcium interaction than bovine milk^[9,21-23]^. While the effects of different casein micelle removal methods on particle characteristics of bovine milk EVs have yet to be determined, our study did not observe major issues for HMEVs in this regard.

Among different methods, chymosin and EDTA obviously shifted the supramolecular structure of casein micelles from the HMEV fractions, where the casein contents were moving to latter fractions, as shown in the dot blot [Figure 2], suggesting the effectiveness of both methods in reducing the size of caseins. Interestingly, proteomic analysis revealed that caseins were still detectable in substantial amounts in the HMEV isolates [Figure 4A]. A possible explanation was that caseins detected in the HMEV isolates were the remnants of sub-micellar caseins, intravesicular proteins, or the surface corona of HMEVs. The surface corona is a set of molecules, mainly proteins, that are adsorbed around EVs in the aqueous phase to constitute the external layer of EVs. The surface corona is thought to affect EV physicochemical properties, cellular uptake, and tissue biodistribution^[31]^. This hypothesis that detected caseins in the HMEV isolates were remnants of the surface corona of EVs was supported by the ratio of caseins to CD9 [Figure 4B and C] in which, per one unit of CD9, caseins were markedly depleted by EDTA, acetic acid, and chymosin treatments (and slightly reduced by sodium citrate method). Since intravesicular caseins should be considerably stable compared to one unit of CD9, our findings did not support this possibility. To distinguish between sub-micellar caseins and caseins of the HMEV corona, a more rigorous strategy to completely remove sub-micellar caseins would be required, for example, a combination of two or three methods with different mechanisms of casein disaggregation/removal. Thereafter, surfaceomics (proteomics of surface proteins) could be applied to obtain the evidence of caseins presented in the HMEV corona. Proper optimizations are needed to establish a successful combination strategy, including the method type, number, and sequence of action, while the cost, time, and workload per sample should be considered a reasonable trade-off. Given the significant roles of the EV corona, HMEV surfaceomics should also be pursued in future studies.

This study had limitations. First, this study compared casein micelle removal methods and applied qEV size exclusion chromatography as the sole EV isolation technique. The methods performed in this study may slightly differ when applied to other EV isolation methodologies. When applying an isolation method other than qEV size exclusion chromatography, some may use our findings to choose a preferred casein micelle removal method and then confirm its performance in the context of a different EV isolation method. Second, this study used frozen biobanked human milk. Due to the limited volume of individual samples, we generated a master pool from four individual milk samples before aliquoting for each arm of the casein removal methods to ensure that the amount of EVs is sufficient and consistent for all comparison conditions. Concerns about the freezing effects of milk samples on EV integrity and yield were addressed by conducting NTA on EVs from both fresh and frozen samples, stored at -80 °C, from the same donor. The results confirmed that a single freeze-thaw cycle did not adversely affect the EVs [Supplementary Figure 3]. Lastly, this study did not include HMEV functionalities in the comparison. Different chemicals that remove caseins may induce changes in HMEV molecular cargos and their functions. Contamination, even at the minute amount, of sodium citrate, EDTA, acetic acid, and chymosin may interfere with HMEV functional studies. Different degrees of casein depletion and HMEV marker enrichment of four casein micelle removal methods may largely contribute to the HMEV potency. Since the findings would be crucial to breastfeeding medicine and HMEV therapeutics, future studies should be pursued to address those issues.

Table 1 summarizes the performance of four casein micelle removal methods based on particle analysis, casein depletion, HMEV enrichment, workload, and turnaround time. This information can guide the preferred method to handle casein contamination in future studies involving HMEV isolation and characterization. From our perspective, chymosin or EDTA treatments are two methods of choice, and their selection may depend on the number of samples and the specific needs of each project.

**Table 1:** The performance of four casein micelle removal methods in this HMEV study

**Casein micelle removal method^a^**	**Particle size and yield**	**Casein depletion**	**HMEV marker enrichment**	**Step of procedure**	**Turnaround time**
1% Sodium citrate	++	+	+/-	1	< 10 min
20 mM EDTA	+++	++	+++	1	< 10 min
1% Acetic acid	+++	++	++	2	> 30 min
1% Chymosin/CaCl_2_	++	+++	++++	2	> 30 min

^a^Final concentration in the mixture; +/-: It indicate a slightly enrichment to unchange; HMEV: Human milk extracellular vesicle.

## DECLARATIONS

### Availability of data and materials

The data that support the findings of this study are available in the manuscript and Supplementary Materials.

### Copyright

© The Author(s) 2024.
