# Supplementary material for: Which casein micelle removal method is suitable for studies of human milk extracellular vesicles? A systematic comparison of four different treatments for casein depletion before extracellular vesicle isolation from human milk
Source: Extracell Vesicles Circ Nucl Acids. 2024 May 22;5(2):221–32. doi: 10.20517/evcna.2024.02 (PMC11648521; doi:10.20517/evcna.2024.02)
Supplement: Supplementary file 1 [file evcna-5-2-221-SupplementaryMaterials.zip › evcna4002-SupplementaryMaterials.pdf]

Supplementary Materials

Which casein micelle removal method is suitable for studies of human milk extracellular vesicles? A systematic comparison of four different treatments for casein depletion before extracellular vesicle isolation from human milk

Hatice Cetinkaya[1,#], Supasek Kongsomros[1,#], Laurie Nommsen-Rivers[2], Ardythe L. Morrow[1], Somchai Chutipongtanate[1,*]

[1]MILCH and Novel Therapeutics Lab, Division of Epidemiology, Department of Environmental and Public Health Sciences, University of Cincinnati College of Medicine, Cincinnati, OH 45267, USA.

[2]Department of Nutrition, University of California Davis, Davis, CA 95616, USA.

[#]Authors contributed equally.

Correspondence to: Dr. Somchai Chutipongtanate, Department of Environmental and Public Health Sciences, University of Cincinnati College of Medicine, 160 Panzeca Way, Cincinnati, OH 45267, USA. E-mail: chutipsi@ucmail.uc.edu

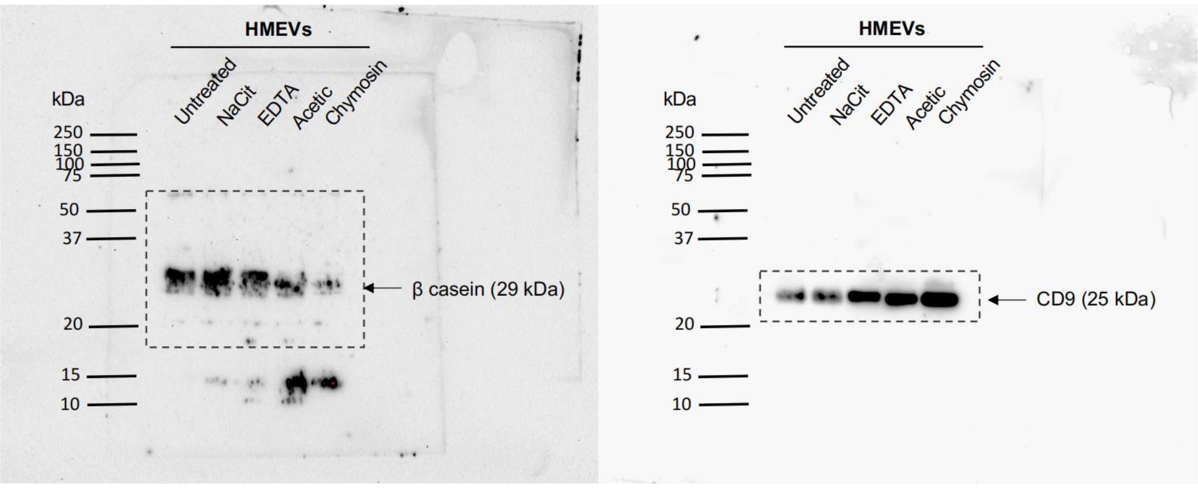

Supplementary Figure 1. Venn diagram illustrated the number of shared and unique HMEV proteins among different casein micelle removal methods (full details of protein names provided in Supplementary Table 3).

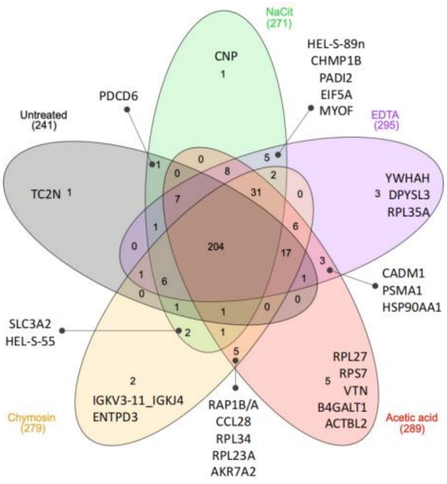

Supplementary Figure 2. The full-length immunoblot results of the cropped images (dash line), as presented in Figure 4C.

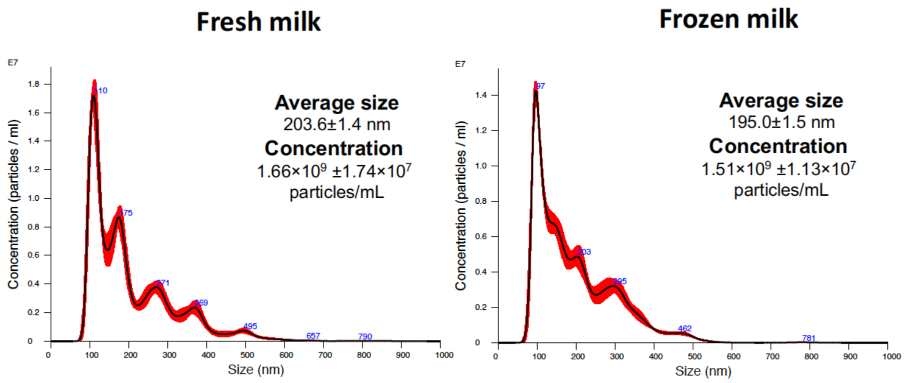

Supplementary Figure 3. Nanoparticle Tracking Analysis of HMEVs from Fresh and Frozen Milk Samples. NTA shows the size distribution, average diameter, and concentration (particles/mL) of human milk extracellular vesicles (HMEVs) isolated from fresh and frozen milk samples, stored at -80°C for 18 hours to make sure that it is frozen, obtained from the same donor.

Supplementary Table 1. Full details of 393 HMEV proteins identified by DDA and BoxCAR proteomics with MaxQuant search.

Supplementary Table 2. Eighty-seven proteins consistently identified by DDA and BoxCAR proteomics

Supplementary Table 3. Lists of shared and unique proteins identified according to the Venn diagram as shown in Supplementary Figure 2.
